# In-hospital cardiac arrest: the state of the art

**DOI:** 10.1186/s13054-022-04247-y

**Published:** 2022-12-06

**Authors:** James Penketh, Jerry P. Nolan

**Affiliations:** 1 Intensive Care Unit, Royal United Hospital, Bath, UK; 2 Warwick Clinical Trials Unit, University of Warwick, Coventry, UK

**Keywords:** Resuscitation, Cardiac arrest, Treatment, Prognostication, Response

## Abstract

In-hospital cardiac arrest (IHCA) is associated with a high risk of death, but mortality rates are decreasing. The latest epidemiological and outcome data from several cardiac arrest registries are helping to shape our understanding of IHCA. The introduction of rapid response teams has been associated with a downward trend in hospital mortality. Technology and access to defibrillators continues to progress. The optimal method of airway management during IHCA remains uncertain, but there is a trend for decreasing use of tracheal intubation and increased use of supraglottic airway devices. The first randomised clinical trial of airway management during IHCA is ongoing in the UK. Retrospective and observational studies have shown that several pre-arrest factors are strongly associated with outcome after IHCA, but the risk of bias in such studies makes prognostication of individual cases potentially unreliable. Shared decision making and advanced care planning will increase application of appropriate DNACPR decisions and decrease rates of resuscitation attempts following IHCA.

## Background

In-hospital cardiac arrest (IHCA) is defined in the Utstein resuscitation registry reporting template as the delivery of chest compressions and/or defibrillation to patients admitted to inpatient beds [1]. In-hospital cardiac arrest is associated with a high risk of death but mortality rates are decreasing [2, 3]. In-hospital cardiac arrest has increasingly been recognised as fundamentally different from out-of-hospital cardiac arrest (OHCA) and is increasingly studied independently from the latter [4]. Consequently, our understanding of IHCA and approach to in-hospital resuscitation continues to evolve.

## Epidemiology and outcome after cardiac arrest

The current reported incidence of IHCA arrest varies between institutions and countries around the world. National cardiac arrest databases have reported rates of IHCA between 1.2 and 9–10 per 1000 admissions. The Danish DANARREST nationwide registry reported a rate of 1.8 per 1000 admissions or 0.6 cardiac arrests per 1000 in-patient days between 2017 and 2018 [5]. In Japan, the incidence of IHCA was recently reported to be 5.1 per 1000 hospital admissions between 2011 and 2017 [6]. Extrapolated data from the Get-With-The-Guidelines-Resuscitation (GWTG-R) registry estimate the incidence of IHCA in the USA to be 9.7 per 1000 hospital admissions [7]. Annual data from the UK National Cardiac Arrest Audit (NCAA) between 2011 and 2021 documents an incidence in the range of 1 and 1.6 per 1000 hospital admissions [8, 9]. Where data have been available for longer, trends in IHCA incidence and outcome can be analysed and these also vary among countries. Rates of IHCA have slightly increased in the USA, whereas in Japan and the UK they have declined, despite an ageing and potentially more co-morbid population. These variations may be explained by global differences in culture (particularly in respect of do-not-attempt cardio pulmonary resuscitation (CPR) decisions), infrastructure and disease.

The IHCA population is now generally recognised as distinct from the OHCA population, most notably in demographics, presenting rhythms and cause of cardiac arrest. However, a recent analysis of the DANARREST dataset suggests there were no substantial differences in demographics, co-morbidities and initial cardiac rhythm, which was predominantly non-shockable, for both IHCA and OHCA patients. The majority of patients were male in both groups and although other co-morbidities were similar, there was a predominance for ischaemic heart disease in the IHCA group [10]. In contrast, a retrospective analysis of patients admitted to the intensive care unit (ICU) after cardiac arrest in Sweden found a predominance of males with higher rates of most co-morbidities including diabetes, heart disease and chronic obstructive pulmonary disease (COPD) [4]. There was also a higher rate of non-shockable rhythms in the IHCA group. It is possible that patients with rapid recovery after treatment of a shockable rhythm may not have needed ICU admission, which might partly account for the predominance of patients with non-shockable rhythms admitted to the ICU. The groups also differ in the number of witnessed events, clinical setting, available personnel and treatment availability. Overall, in comparison with OHCA patients, IHCA patients have higher 30-day survival (24% vs. 17%) [4] and (50.2% vs. 47.8%) [10]. The IHCA patients had better long-term functional survival as determined by CPC scores; however, in multivariable analysis, location of cardiac arrest was not an independent predictor of functional outcome [4].

After IHCA, the chances of 1 year survival without hypoxic ischaemic brain injury or the need for ongoing care increase with younger age, shockable rhythm, shorter time to defibrillation and a lower incidence of COPD. Counterintuitively, these chances increase with a higher rate of heart disease, presumably because of a predominance of shockable rhythms associated with heart disease. Among 30-day survivors, the rate of survival at 1 year without hypoxic ischaemic brain injury is as high as 79.8% [11]. Overall, 1-year survival appears to be improving over time. Observational data from the GWTG-R from 2000 to 2011 suggest an increase in 1-year survival from 4.7 to 10.2% for non-shockable IHCA [3]. A meta-analysis of 40 studies documented a 1-year survival after IHCA of 13.4% [12]. In this meta-analysis, 17.6% of patients survived to hospital discharge, indicating that 76% of patients who survive their hospital stay will survive for at least 1 year [12]. Our understanding of the epidemiology of IHCA is improving but is limited by the observational nature of the data and sampling or selection bias because of incomplete datasets. The expansion of IHCA registries and ability to collect larger datasets consistently will help improve this knowledge.

**Fig. 1:**
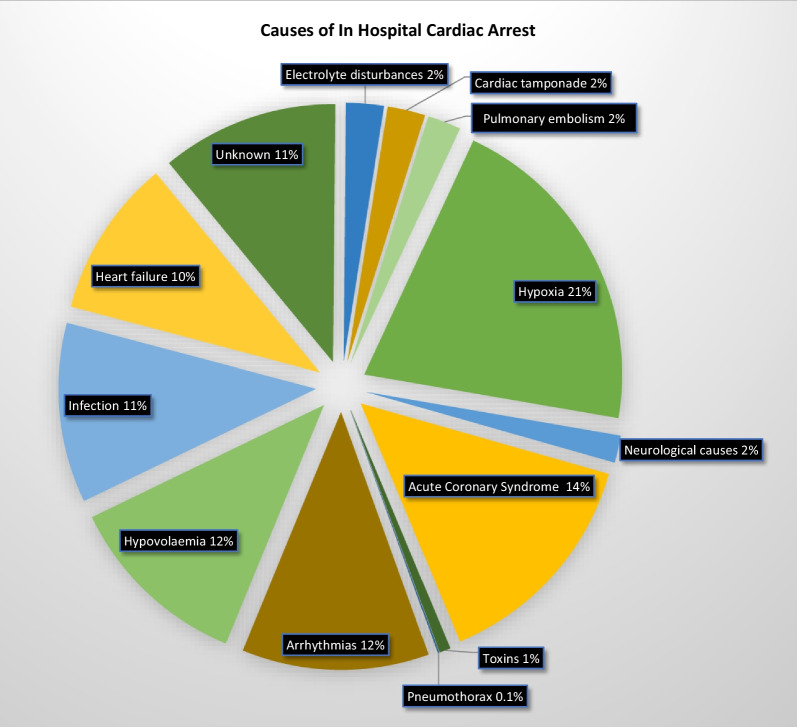
Causes of in-hospital cardiac arrest derived from a large systematic review and meta-analysis [13]. Cause with associated 95% confidence intervals: hypoxia (14.2%–38.7%), neurological causes (1–3.4%), acute coronary syndrome (13.9–22.6%), toxins (0.2–1.6%), pneumothorax (0.06–0.14%), arrhythmias (0–34.9%), hypovolaemia (7.0– 22.7%), infection (9.5–19.3%), heart failure (6.5–18.8%), unknown (5.1–23.2%), electrolyte disturbances (0.9–5.1%), cardiac tamponade (0.3–5.7%), pulmonary embolism (2.2–3.1%)

The established teaching on advanced life support courses is to consider the reversible causes of IHCA in terms of the Hs and Ts. However, a recent systematic review and meta-analysis that included 27,102 patients documented the most prevalent causes of IHCA to be hypoxia, acute coronary syndrome, hypovolemia, arrhythmias, infection and heart failure [13] (see Fig. 1). Three of these (arrhythmia, infection and heart failure) are not included among the traditional Hs and Ts.

## In-hospital cardiac arrest registries

The first Utstein-style reporting guideline for IHCA was published in 1997 [14]. The guideline was designed to enable reliable comparison of the characteristics, trends in interventions and outcomes from IHCA between hospitals, regions and countries [15, 16]. Twenty years of standardised reports and outcome data of IHCA have been available since its publication. Several worldwide databases using the Utstein-style guidelines have been established and provide valuable insight into IHCA [5, 6, 9, 17, 18]. The Utstein-style guideline for IHCA was updated in 2019 [1]. High-quality IHCA registries are powerful resources that through observational studies can assess resuscitation strategies and guidelines [19, 20].

The number of IHCA registries has recently increased following publication of the first reported national IHCA registry in China, the BASeline Investigation of Cardiac Arrest (BASIC)–IHCA [21]. The BASIC-IHCA registry includes 40 hospitals across 29 of the 31 Chinese provinces and has documented 19,443 IHCAs after 18 months of data collection. The data align with Utstein guidelines providing valuable insights into IHCA in China and enabling international comparisons with cardiac arrest strategy. Some of planned data to be collected, such as functional status at 6 and 12 months, are outside of the Utstein dataset but may be particularly informative. This registry will hopefully stand alongside existing registries to further our understanding of IHCA.

Existing international registries such as the US GWTG-R registry and the UK NCAA have provided data that have informed and supported cardiac arrest guidelines [22]. Furthermore, they have enabled us to understand how the incidence of cardiac arrest is influenced by guidelines and medical advances [23]. Cardiac arrest registries are a valuable resource enabling IHCA research and their continued development is strongly encouraged.

## Cardiac arrest response

The in-hospital response to cardiac arrest has developed over time and varies between institutions. Most commonly, IHCA now triggers the presence of a team of specially assigned first responders. These teams may be dedicated only to caring for patients in cardiac arrest or more broadly for peri-arrest or critically ill patients and are sometimes referred to as medical emergency teams (MET) or rapid response teams (RRT) [24, 25]. However, cardiac arrest/MET teams are not universal and IHCA patients may be treated by location specific teams [21]. The introduction of RRTs has been attributed to creating a downward trend in hospital mortality since their introduction [25].

Despite standardisation of practice across many countries, survival rates following IHCA vary internationally. Logically, the algorithmic approach to resuscitation should result in similar outcomes between centres after adjustment for patient factors; however, significant variation remains. One proposed reason for the variation in outcome is the implementation of resuscitation guidelines. The design of cardiac arrest teams has been reported as a key factor in cardiac arrest performance [23]. Dedicated cardiac arrest teams where the sole member responsibility was to respond as part of the arrest team or where clinical duties enabled quick redirection of focus to the cardiac arrest team are noted to be a feature of centres with higher IHCA survival [26]. Other features of high-achieving hospitals include pre-allocation of specific roles, inclusion of trained and/or experienced personnel, and good communication between team members. Training is an important factor, specifically the fidelity of mock resuscitation, with more unplanned, in situ multidisciplinary simulations appearing to enhance performance [26].

Whilst evaluating guideline recommended process measures, one study has documented that each 10% increase in hospital process composite performance was associated with a 22% higher adjusted odds of survival [adjusted adds ratio (OR), 1.22; 95% confidence interval (CI) 1.08–1.37; *P* = 0.01] [20]. Hospital process composite performance was assessed through adherence with guideline process measures; device confirmation of correct tracheal tube placement, a monitored or witnessed cardiac arrest, time to first chest compression ≤ 1 min, time to first defibrillation delivered ≤ 2 min for VF/VT and administration of adrenaline or vasopressin for within 5 min pulseless events [20]. When measured by process composite performance, the highest achieving hospitals have greatest adherence to individual guideline measures for IHCA. GWTG-R data show that adherence with confirmation of tracheal tube positioning was 70.8% in quartile 1 (lowest process composite performance) to 94.3% in quartile 4 (highest process composite performance, *P* = 0.01 [20]. Hospitals that achieved higher rates of first defibrillation at < 2 min also had higher process composite scores (49.4% in quartile 1 to 66.5% in quartile 4, *P* = 0.01) [20]. These data highlight the importance of certain processes within the package of IHCA care. It therefore follows that recording and tracking key performance indicators may drive up overall performance and improve patient care although wider systems processes may also influence results. Indeed, measuring and tracking immediately quantifiable elements of the cardiac arrest team performance is associated with improved patient survival [20]. After multivariate adjustment, hospitals that track interruptions in chest compressions are twice as likely to be part of a higher-surviving quintile centre compared with peers that do not track [19]. Monitoring times to defibrillation similarly improved risk standardised survival rates for IHCA [19]. Higher-performing hospitals further review their cardiac arrest data more frequently, with all upper quintile hospitals reviewing data at least quarterly [19]. The perception of adequate training by resuscitation team members improved odds ratio of being a higher-surviving centre by 3 [19].

## Treatment

There have been no major changes to treatment recommendations in the latest treatment consensus guidelines [27]. Defibrillation remains a cornerstone of modern cardiac arrest management. Technology and access to defibrillators continues to progress. Analysis of cardiac arrest registry data informs us about trends in defibrillation strategy. The use of deferred repeat shocks (> 1 min after the initial shock instead of ‘stacked’ shocks), which was first recommended in the 2005 resuscitation guidelines [28], has increased from 26 to 57% between 2005 and 2012 [29]. Deferred defibrillation was not associated with survival to hospital discharge (adjusted risk ratio (RR) 0.89, 95% CI 0.78 to 1.01; *P* =  0.08). Nevertheless, the overall survival rates for in hospital VF/VT arrest have improved, perhaps reflecting the wider strategies in cardiac arrest care such as minimising interruptions in chest compressions in IHCA patients with persistent arrhythmias [30].

## Airway

The question of the optimal method for ensuring airway patency during IHCA remains unanswered and is considered a specific knowledge gap by the International Liaison Committee on Resuscitation (ILCOR) [31]. Considerations for the choice and timing of an optimal airway device include how to minimise interruptions in chest compressions, the skills required for insertion, optimising cerebral perfusion and how failed placement may affect overall outcome. Large, randomised trials have to date focused on the prehospital population where it appears there is no significant functional outcome benefit when comparing bag-mask ventilation, supraglottic airways (SGAs) and tracheal tubes [32–34]. Observational studies suggest that there is wide variation in the chosen airway devices used in IHCA between treating centres [35, 36]. Intubation rate ranged between 21 and 90%, whilst supraglottic airway use varied between 1 and 45% [36]. In a multinational cohort of 598 patients, 30% were managed without advanced airway, 54% had tracheal intubation, and 17% had a supraglottic airway [36]. Evidence from the US GWTG-R registry indicates that rates of tracheal intubation during IHCA are decreasing [37]. Understanding any benefit to a choice of IHCA airway will be helped by future randomised control trials, such as Airways-3 (a strategy of tracheal intubation versus supraglottic airway during IHCA) which is about to start enrolling patients throughout the UK.

## Extracorporeal CPR

The use of extracorporeal CPR (ECPR) via the application of veno-arterial extracorporeal membrane oxygenation (ECMO) has increased substantially [38]. ECPR is acknowledged in ILCOR guidelines as an option for select patients with reversible pathology (weak recommendation, very low-certainty evidence) [27]. Retrospective observational data suggest that ECPR is a feasible with reported survival to hospital discharge as high as 34% in patients receiving > 10 min of CPR [38]. We are not aware of any published randomised trials of ECPR for IHCA patients, likely because of the ethical, practical and economical challenges associated with such trials. Potential outcome benefits of ECPR in IHCA patients have been suggested for selected patients from observational studies. Improved survival to discharge and 30 day functionally favourable survival have been reported for patients receiving ECPR following matched pair analysis against conventional CPR alone [38]. The case for ECPR is not straightforward as providing ECPR for the IHCA population currently requires substantial investment in training, resources, personnel and organisation [38]. Furthermore, ECPR has the potential to provide organ support to patients who may have no meaningful chance of recovery, creating ethical dilemmas for some. It is also associated with a high economic cost [38]. Considering the challenges of ECPR, careful patient selection appears essential, at least until further high-certainty data are available.

## Temperature control after IHCA

Therapeutic hypothermia or targeted temperature management (TTM) has been the subject of much research into post-resuscitation care over the past decade, but most randomised trials have focussed on OHCA. The 2022 ILCOR consensus on CPR science recommends that the term ‘temperature control’ be used to prevent conflation with the specifically named TTM studies [27]. The recent HYPERION study recruited patients with ROSC from non-shockable rhythms after both IHCA and OHCA. The trial demonstrated an increase in favourable CPC score (1–2) at day 90 (10.2% vs. 5.7%, difference 4.5% (95% CI 0.1–8.9), *p* = 0.04) in patients who were cooled to 33 °C (± 0.5 °C) compared with the normothermic control group [39]. Variations in the time and depth of sedation, the presence of fever in > 5% of the control group and a low fragility index mean the case for hypothermia in IHCA patients is not established with high-certainty. A secondary analysis of the HYPERION study that focused on the IHCA patients showed that temperature control at 33 °C for 24 h was associated with a higher percentage of patients with favourable 90-day cerebral performance category (CPC) scores compared with the normothermic group (16.4% vs. 5.8%; *P* =  0.03) [40]. Current guidelines from the European Resuscitation Council and European Society of Intensive Care Medicine recommend actively preventing fever (defined as a temperature > 37.7 °C) in post-cardiac arrest patients who remain comatose (weak recommendation, low-certainty evidence) [41].

## Prognostic factors associated with survival after IHCA and early prognostication

Prognostication before, during and after cardiac arrest can be challenging. Understanding the prognostic factors associated with IHCA may help to determine outcomes for patients before and after the event and can also guide important areas of care and future research.

A meta-analysis of adjusted results from 23 studies, including a large patient cohort of more than 90,000 patients, identified several pre-arrest factors that are associated with decreased survival [42]. Male sex (pooled OR 0.84; 95% CI = 0.73 to 0.95), increasing age (70 and older = pooled OR of 0.42 (95% CI0.18 to 0.99), active malignancy (pooled OR 0.57; 95% CI 0.45 to 0.71) and renal disease (pooled OR 0.56; 95% CI = 0.40 to 0.78) are associated with decreased survival from IHCA. Whilst understanding these non-modifiable risk factors could build a picture of those who may be less likely to achieve short term (28–30 day) survival, the retrospective and observational nature of these studies may generate substantial bias.

The same meta-analysis demonstrated several intra arrest factors which were associated with increased survival [42]. Increased survival for witnessed IHCA (pooled OR 2.71; 95% CI 2.17 to 3.38), arrests in monitored settings (OR 2.23; CI 1.41 to 3.52) and IHCA during times when hospitals are fully staffed (OR of 1.41 (CI 1.20 to 1.66) suggest system processes may be modifiable to improve outcomes. Resuscitation attempts longer than 15 min (OR 0.12; CI 0.07–0.19) and intubation during cardiac arrest (OR 0.54; CI 0.42–0.70) were associated with decreased survival [42]. Given the observational nature of these data, and without knowledge of clinical decisions influencing them, no clear conclusions can be drawn. However, these are areas that further prospective trials could focus on. The decrease in survival associated with tracheal intubation is again potentially associated with bias but is consistent with a previous analysis of the US GWTG-R registry [43]. Upcoming randomised controlled trials, such as Airways-3, will hopefully provide greater insight into the effect of airway choice during IHCA on outcome.

Frailty has been defined as reduced physiological reserve and vulnerability to adverse health outcomes from physiological stressors which result from the accumulation of age and disease related deficits [44]. Several studies have shown an association between frailty and worse outcome after IHCA [45–47]. Retrospective analysis of 477 patients between 2013 and 2016 concluded worse outcomes for those with a clinical frailty score of 5 or more [45]. Frailty was independently associated with lower odds of ROSC (adjusted OR 0.63 [95% CI 0.41–0.93]) and higher odds of mortality (adjusted OR 2.91 [95% CI: 2.37–3.48]). Not only were absolute outcomes worse for frail patients but they were more likely to be discharged to a long-term care facility (adjusted OR 1.93 [95% CI 1.57–2.32]) [45]. Pooled results from meta-analysis found the presence of frailty was associated with an approximate three-fold increase in the odds of dying in-hospital after IHCA (adjusted OR = 2.93; 95% CI = 2.43–3.53)[46]. Understanding the impact of frailty on outcome is another part of the puzzle informing outcomes and prognosis after IHCA.

There is some evidence that the quality of care during IHCA has improved in recent years [48], but despite this only approximately 1 in 5 patients survive to hospital discharge. Do-not-attempt CPR (DNACPR) and treatment escalation plans (TEPS) enable clinicians and patients to make forward planning decisions about resuscitation plans before cardiac arrest occurs [49]. Age and co-morbidities may help frame these decisions, but quantification and prediction of meaningful outcomes is challenging.

The GO FAR 2 score is a prognostic scoring system validated for predicting survival following an IHCA with a CPC score ≤ 2 [50]. Likelihood of CPC ≤ 2 is predicted as very poor (≤ 5%), poor (> 5–10%), below average (≤ 10%), average (> 10–30%) and above average (> 30%). The model was developed using data from the GWTG-R dataset using a least absolute shrinkage and selection (LASSO) model. Univariate analysis suggests age > 85 (-12.1%), admission CPC < 2 (-14.4%) and non-cardiac surgical admission (-18.8%) had the largest negative percentage difference for survival with CPC ≤ 2. Data analysis was limited to variables included in the GWTG-R registry and follow-up was missing for some patients which may introduce bias. The score does not take into account individual hospital performance so may over or underestimate depending on survival rates in individual hospitals. Nevertheless, the score is a useful tool to initiate the decision-making process and a valuable tool in addition to clinical judgement.

## Do-not-attempt cardiopulmonary resuscitation and emergency care treatment plans

European Resuscitation Council guidelines promote the use of integrated documentation combining DNACPR decisions with emergency care treatment plans (ECTP) [30]. Emergency care treatment plans document patient preferences and clinicians’ treatment recommendations to guide those making decisions in an emergency and when a patient may not have the capacity to be involved in such decisions [49]. These documents are a progression from single purpose DNACPR forms developed in the 1970s. Nationwide standardised ECTPs, termed Recommended Summary Plan for Emergency Care and Treatment (ReSPECT), have been established in the UK through iterative design and stakeholder consultation [49]. The form is designed to be used across all healthcare settings and promotes advanced care planning through shared decision making. Evaluation of its use and impact on health outcomes are awaited.

The promotion of shared decision making and advanced care planning, combined with increases in proactive appropriate DNACPR decisions, may partly explain the decreasing rates of IHCA in some countries [6, 8, 9].

## Conclusion

In-hospital cardiac arrest creates a significant burden for healthcare facilities and continues to be associated with a high mortality rate. Progress continues to be made in our approach to its treatment through use of data from observational analyses of IHCA registries and prospective trials, and increasingly selective resuscitation attempts. We must continue to ensure that the IHCA population is not denied state-of-the-art resuscitation care.

## Data Availability

Not applicable.

## Abbreviations

BASIC: BASeline Investigation of Cardiac Arrest
CPC: Cerebral performance category
COPD: Chronic obstructive pulmonary disease
CPR: Cardio pulmonary resuscitation
DNACPR: Do-not-attempt CPR
ECTP: Emergency care treatment plans
ECMO: Extracorporeal membrane oxygenation
ECPR: Extracorporeal CPR
GWTG-R: Get-With-The-Guidelines-Resuscitation
IHCA: In-hospital cardiac arrest
ILCOR: International Liaison Committee on Resuscitation
LASSO: Least absolute shrinkage and selection
MET: Medical emergency teams
NCAA: National Cardiac Arrest Audit
OR: Odds ratio
OHCA: Out-of-hospital cardiac arrest
RRT: Rapid response teams
ReSPECT: Recommended Summary Plan for Emergency Care and Treatment
RR: Risk ratio
SGAs: Supraglottic airways
TEPS: Treatment escalation plans
TTM: Targeted temperature management
TTM: Targeted temperature management
